# Supplementary material for: Genome-Wide Identification and Expression Profiling of Dehydration-Responsive Element-Binding Family Genes in Flax (Linum usitatissimum L.)
Source: Int J Mol Sci. 2025 Mar 27;26(7):3074. doi: 10.3390/ijms26073074 (PMC11988780; doi:10.3390/ijms26073074)
Supplement: Supplementary file 1 [file ijms-26-03074-s001.zip › Supplementary Figures.pdf]

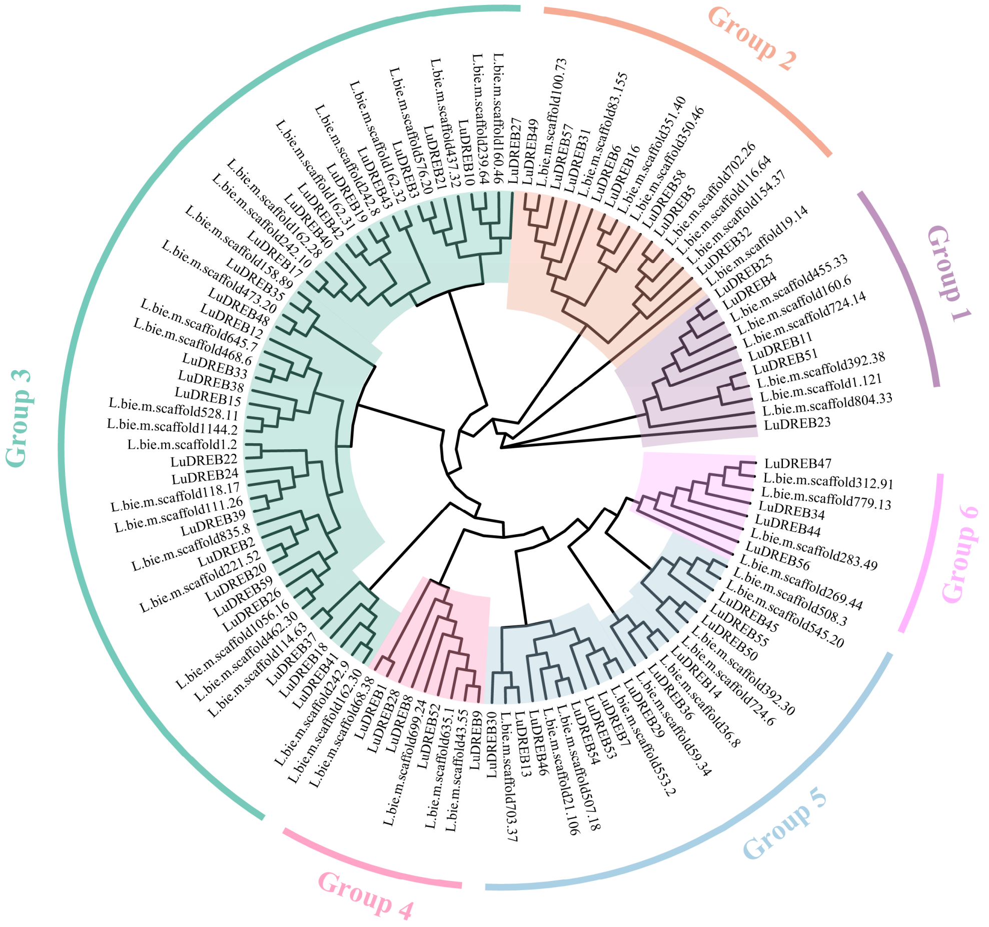

Figure. S1 Phylogenetic tree of DREB proteins from Longya-10 and pale flax. The tree is divided into six clades, each represented by a different color and designated as Group 1 to Group 6.
